# *In vivo* transduction of neurons with TAT-UCH-L1 protects brain against controlled cortical impact injury

**DOI:** 10.1371/journal.pone.0178049

**Published:** 2017-05-24

**Authors:** Hao Liu, Marie E. Rose, Xiecheng Ma, Sherman Culver, C. Edward Dixon, Steven H. Graham

**Affiliations:** 1 Geriatric Research Educational and Clinical Center, V.A. Pittsburgh Healthcare System, Pittsburgh, Pennsylvania, United States of America; 2 Department of Neurology, University of Pittsburgh School of Medicine, Pittsburgh, Pennsylvania, United States of America; 3 Department of Neurosurgery, University of Pittsburgh, Pittsburgh, Pennsylvania, United States of America; 4 Department of Critical Care Medicine, University of Pittsburgh, Pittsburgh, Pennsylvania, United States of America; University of Florida, UNITED STATES

## Abstract

Many mechanisms or pathways are involved in secondary post-traumatic brain injury, such as the ubiquitin-proteasome pathway (UPP), axonal degeneration and neuronal cell apoptosis. UCH-L1 is a protein that is expressed in high levels in neurons and may have important roles in the UPP, autophagy and axonal integrity. The current study aims to evaluate the role of UCH-L1 in post-traumatic brain injury (TBI) and its potential therapeutic effects. A novel protein was constructed that fused the protein transduction domain (PTD) of trans-activating transduction (TAT) protein with UCH-L1 (TAT-UCH-L1) in order to promote neuronal transduction. The TAT-UCH-L1 protein was readily detected in brain by immunoblotting and immunohistochemistry after i.p. administration in mice. TBI was induced in mice using the controlled cortical impact (CCI) model. TAT-UCH-L1 treatment significantly attenuated K48-linkage polyubiquitin (polyUb)-protein accumulation in hippocampus after CCI compared to vehicle controls, but had no effects on K65-linkage polyUb-protein. TAT-UCH-L1 treatment also attenuated expression of Beclin-1 and LC3BII after CCI. TAT-UCH-L1-treated mice had significantly increased spared tissue volumes and increased survival of CA3 neurons 21 d after CCI compared to control vehicle-treated mice. Axonal injury, detected by APP immunohistochemistry, was reduced in thalamus 24 h and 21 d after CCI in TAT-UCH-L1-treated mice. These results suggest that TAT-UCH-L1 treatment improves function of the UPP and decreases activation of autophagy after CCI. Furthermore, TAT-UCH-L1 treatment also attenuates axonal injury and increases hippocampal neuronal survival after CCI. Taken together these results suggest that UCH-L1 may play an important role in the pathogenesis of cell death and axonal injury after TBI.

## Introduction

Traumatic brain injury (TBI) is often associated with diffuse axonal pathology that leads to significant motor and cognitive deficits [1, 2]. Although mechanical forces can shear and break axons, intact axons may also be injured due to impaired axonal transport resulting in axonal swelling, varicosities and bulbs that develop within hours after TBI [2]. This axonal injury after TBI is associated with accumulation of ubiquitinated (Ub)-proteins and amyloid precursor protein (APP) in damaged axons [3, 4]. The ubiquitin proteasome pathway (UPP) tags damaged or misfolded proteins for degradation and dysfunction of the UPP and thus may contribute to axonal injury and recovery after TBI [5]. There are no proven effective therapies that ameliorate failure of the UPP or axonal injury after TBI.

The UPP is composed of numerous components which include ubiquitin, the 26S proteasome, ubiquitin activationg enzyme (E1), ubiquitin conjugating enzyme (E2), ubiquitin ligating enzyme (E3), and deubiquitinating enzymes (DUBs) [6]. As a unique DUB with ligase activity [7], ubiquitin carboxy-terminal hydrolase L1 (UCH-L1) is a multifunctional neuronal protein that has been associated with the UPP in neurons, axonal transport and integrity, and cell survival [8–15]. Mutation or disruption of UCH-L1 is associated with many neurodegenerative diseases such as Alzheimer’s disease and Parkinson’s disease, which are characterized by severe Ub-protein accumulation, axonal injury and neuron degeneration [8, 15, 16]. Increased UCH-L1 has been observed after spinal cord transection and spinal cord interneurons with increased UCH-L1 levels are more resistant to traumatic injury-induced cell death [10, 17, 18]. We hypothesized that UCH-L1 may play a role in maintaining UPP function, preventing cell death and maintaining axonal integrity in neurons after TBI. To test this hypothesis, a protein that fuses the protein transduction domain (PTD) of trans-activating transduction (TAT) protein with UCH-L1 (TAT-UCH-L1) was constructed that effectively transduces neurons when given systemically *in vivo*. TBI was induced in mice using the controlled cortical impact (CCI) model. The effect of TAT-UCH-L1 or vehicle upon Ub-protein accumulation, hippocampal neuron cell survival and axonal injury after TBI was determined. These studies address whether UCH-L1 expression is an important determinant of cell survival and axonal injury after TBI and whether TAT-UCH-L1 has potential as a novel therapeutic strategy in TBI treatment.

## Materials and methods

This study was carried out in strict accordance with the recommendations in the Guide for the Care and Use of Laboratory Animals of the National Institutes of Health. The protocol was approved by the University of Pittsburgh Institutional Animal Care and Use Committee (Protocol Number: IS00001941). All surgery was performed under isoflurane anesthesia, and all efforts were made to minimize suffering. Animals were housed in a temperature and humidity controlled environment with 12 h light cycles and free access to food and water.

### Reagents and antibodies

Mouse monoclonal anti-poly-ubiquitinated conjugates antibody (clone FK1) were obtained from Enzo Life Sciences (Plymouth Meeting, PA), anti-ubiquitin (linkage-specific K63) antibody was from Abcam (Cambridge, MA) and anti-ubiquitin (K48-specific) was from Millipore (Temecula, CA). Anti-NeuN antibody was from Millipore; Anti-HA, anti-LC3B and anti-Beclin-1 rabbit antibodies were from Cell signaling technology (Boston, MA). Monoclonal anti-GAPDH and anti-HA antibodies were from Covance (Berkeley, CA); Cy3-conjugated monoclonal mouse anti-biotin and Alexafluor 488-conjugated secondary antibodies were from Jackson Immunoresearch Lab (West Grove, PA). Anti-β-actin antibody and all other chemicals were from Sigma-Aldrich.

### Generation and purification of TAT-UCH-L1

The DNA sequence encoding full-length mouse UCH-L1 was amplified by PCR, and cloned into a modified pET30a vector containing the N-terminus PTD of TAT protein and HA sequences (provided by Drs. Jun Chen and Guodong Cao, University of Pittsburgh) [19]. Constructs were confirmed by sequencing.

Plasmids were introduced into Escherichia coli Rosetta (DE3) strains (Novagen, San Diego, CA) producing 6X His-tagged TAT-UCH-L1. Protein production was induced by shaking the E. coli Rosetta cells in medium containing 0.8mM Isopropyl β-D-1-thiogalactopyranoside (IPTG) at 35°C for 4h before harvest by centrifugation. Cell pellets were then lysed and purified with a QIAexpress Ni-NTA Fast start kit (Qiagen, Hilden, Germany). Purified protein was dialyzed against 1XPBS buffer and protein concentration was determined by BCA protein assay (Pierce). Protein purity was assessed by SDS-PAGE and Coomassie blue staining.

### TAT-UCH-L1 injection and traumatic brain injury (TBI)

Animal studies were conducted according to the National Institutes of Health Guide for the Care and Use of Laboratory Animals and with approval of the University of Pittsburgh Institutional Animal Care and Use Committee. Male mice (C57BL/6J, aged 10 wks, 22- 25g) obtained from The Jackson Laboratory (Bar Harbor, ME), were housed in a humidity and temperature controlled environment with free access to food and water and 12 h light cycles. The controlled cortical impact (CCI) model of traumatic brain injury used in this study was performed as described in detail previously[20, 21]. Briefly, mice were anesthetized using 5% isoflurane in 60% nitrous oxide, balance oxygen, and then placed in a stereotaxic frame (David Kopf Instruments, Tjunga, CA) fitted with a nosecone. Isoflurane was lowered to 1–2% isoflurane in the same carrier gas mixture and the head was shaved and prepped with povidone-iodine. A midline incision was made, the skin retracted, and a 5 mm craniectomy was performed over the right parietotemporal cortex. The impactor tip (3 mm) was zeroed to the surface of the brain prior to injury (impact speed: 6 m/sec, injury depth: 1.6 mm, dwell time: 50 msec, angle of impact 20^o^). Injury settings used were designed to generate underlying cortical and hippocampal tissue loss and were consistent with previous studies. Body temperature was maintained at 36.5–38.0^°^C using a warming pad. Following TBI, the incision was closed and anesthesia was discontinued. Bupivacaine was applied topically to the wound for pain relief. Mice remained on the warming pad until recovered from anesthesia then were returned to their home cage. Sham surgery was identical to that described above without trauma. After randomization, mice were administered 60 mg/kg TAT-UCH-L1 or PBS as vehicle control via intraperitoneal injection 1h prior to CCI or sham surgery. The TAT-UCH-L1 dose was determined based on preliminary experiments detecting the TAT HA tag in brain at various timepoints and concentrations (data not shown). Mice were monitored daily post injury. Animals exhibiting signs of infection (redness, swelling or discharge) or pain (hunched posture, freezing, or vocalization) or weight loss greater than 20% of controls were removed from the study and euthanized by carbon dioxide inhalation.

### Brain tissue preparation and Western blotting

Pericontusional brain cortex and hippocampus were dissected from mice at 1h, 4h or 24h after TAT-UCH-L1 treatment and injury and rapidly frozen on dry ice until homogenization with T-PER tissue protein extraction reagent (Pierce) supplemented with protease and phosphatase inhibitors. Protein concentrations were measured by BCA assay. Western blotting was performed as previously described [22]. For LC3B, Beclin-1 and HA detection, cell lysates were resolved on 10% or 12% SDS-PAGE. For Ub-protein detection, cell lysates were resolved on a 4–20% linear gradient polyacrylamide gel (BioRad, Hercules, CA) before incubation with anti-poly-ubiquitinated conjugates, anti-ubiquitin K48-specific or anti-ubiquitin K63-specific antibodies (1:1000 for all). Blots were washed and the appropriate secondary antibodies applied. Protein signal was visualized with ECL reagents (Pierce). Blots were also probed using anti-GAPDH or β-actin antibodies for verification of equal protein loading. N = 5 per group. Densitometric analysis was performed using ImageJ 1.50i software and results are normalized to the corresponding contralateral band.

### Cell survival measurement

Mice were anesthetized with 4% isoflurane in N_2_O/O_2_ (2:1) and transcardially perfused with 20 mL of heparinized saline (4 u/ml) followed by 20 mL of 10% buffered formalin 21 days post TBI. Whole brains were embedded in paraffin and 7 μm thick serial sections were collected every 1 mm. Sections were stained with hematoxylin and eosin and hippocampi were examined using 20X brightfield magnification. All morphologically normal neurons (those with nuclear and cytoplasmic staining) in ipsilateral and contralateral CA1, CA3 and CA4 regions at Bregma -2.2 mm (center of the contusion) were counted by a blinded observer [23]. Data are expressed as percent contralateral. N = 6 per group.

### Assessment of spared tissue volume

Spared tissue volume was calculated using the method of Swanson et al by measuring surviving brain tissue in ipsilateral and contralateral hemispheric areas at each slice using ImageJ 1.50i software. Spared volume was determined by multiplying slice area by slice interval thickness then adding together all slices[24, 25]. Spared tissue volume is expressed as percent contralateral and is calculated as follows: ipsilateral / contralateral * 100. N = 12–15 per group.

### Fluorescent immunohistochemistry/ APP immunostaining assessment

Amyloid precursor protein (APP) immunofluorescent staining was performed as previously described [21, 26]. Mice were sacrificed 24 h and 21 D after injury and brain sections taken at the center of the lesion were immunostained with anti-beta-APP Antibody (1:700, Invitrogen, 51–2700,CT695 or Millipore MAB348) then incubated with AlexaFluor 488-conjugated goat anti-rabbit secondary antibody. Sections were photographed using an Olympus BX51 microscope and Stereo Investigator software (MBF Bioscience, Williston, VT). APP positive cells were counted in 1400 μm^2^ fields from 20X fluoromicrographs located in thalamus (-2.2 mm from Bregma). N = 5 animals per group. Brain sections incubated without application of primary antibody served as controls.

### Statistical analysis

Data are expressed as means +/- SE. Cell counting and spared tissue volumes were analyzed using Student’s T test; Densitometric comparison of immunoblots and APP-positive neurites were analyzed using ANOVA with Dunnett’s or Bonferroni post hoc analysis, respectively, using IBM SPSS statistical software. Results were considered to be significant when p < 0.05.

## Results

### Generation of TAT-UCH-L1 fusion protein

**Fig 1:**
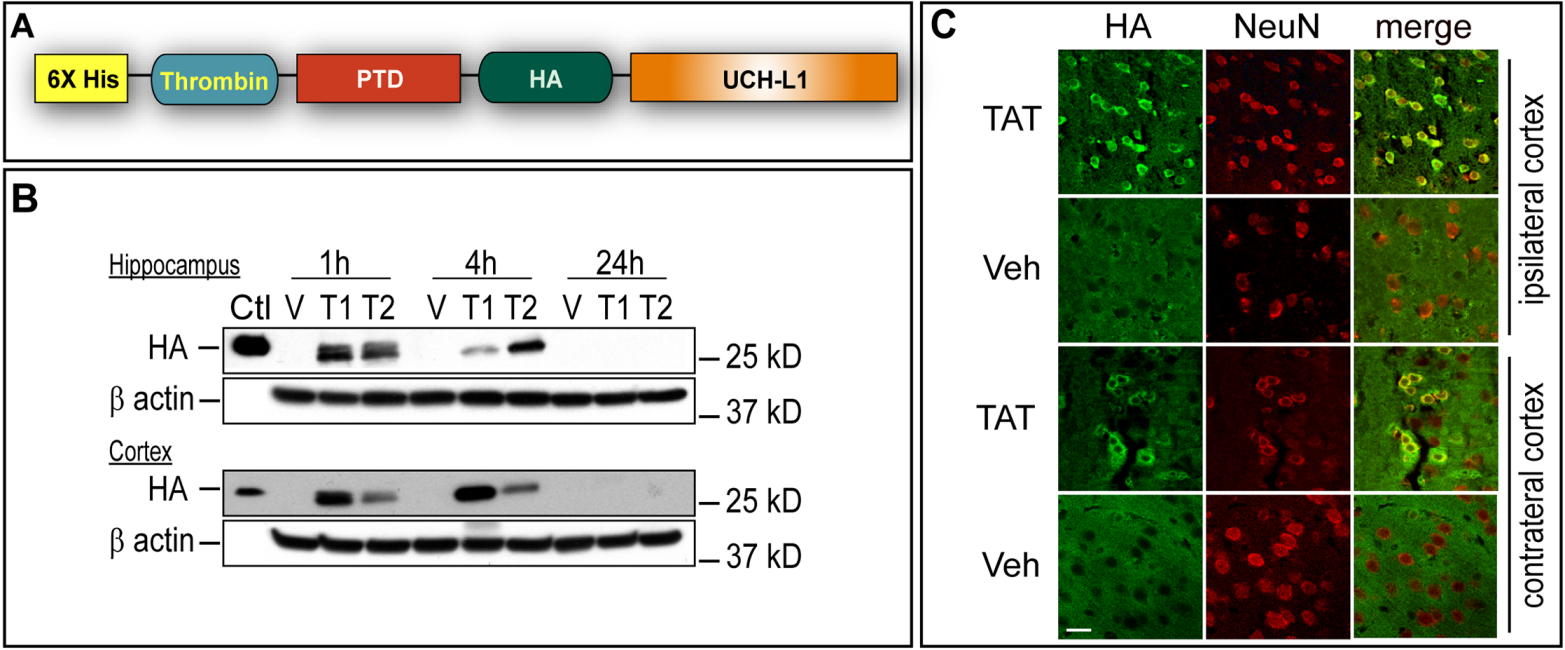
Construction TAT-UCH-L1 fusion protein and detection of neuronal transduction *in vivo*. **A.** Structure diagram of HA-tagged TAT-UCH-L1 fusion protein. **B-C**. Detection of TAT-UCH-L1 fusion protein after CCI. Mice were injected i.p. with TAT-UCH-L1 fusion protein (T, 60 mg/kg) or vehicle (V) and sacrificed 1 h, 4 h, and 24 h post injury. B. Immunoblots of mouse brain hippocampus and cortex using anti-HA antibody. Ctl: recombinant TAT-UCH-L1 protein. C. Immunohistochemical detection of TAT-UCH-L1 fusion protein at 4 h post injury in cortex using anti-HA (green) and anti-NeuN (red) antibodies. Bar = 20μm.

To construct a neuron-permeable form of UCH-L1 that readily transduces neurons *in vivo*, a TAT-UCH-L1 wild type (WT) fusion protein was generated. In addition to the PTD sequence and UCH-L1, TAT-UCH-L1 also contains a 6X His tag for purification and an HA tag for detection (Fig 1A)[19]. Construction and expression of the fusion protein was confirmed by sequencing (S1 Fig, Genomics and Proteomics Core Lab, University of Pittsburgh), and immunoblotting using both anti-UCH-L1 [19], and anti-HA antibodies (Fig 1). TAT-UCH-L1 protein function was also confirmed by performing an *in vitro* hydrolase activity assay; TAT-UCH-L1 WT and TAT-UCH-L1 C90S fusion proteins exhibited comparable hydrolase activities to their respective native UCH-L1 proteins (S2 Fig).

The ability of TAT-UCH-L1 to transduce into mouse brain was evaluated by immunoblotting and immunostaining. Mice were injected with 60mg/kg i.p. of TAT-UCH-L1 or vehicle and sacrificed at 1 h, 4 h and 24 h post CCI. As shown in Fig 1B, TAT-UCH-L1 was detectable by immunoblotting in both hippocampus and cortex of mouse brain at 1 h and 4 h but was not reliably detected at 24 h post injury. Some variability in the amounts of TAT-UCH-L1 protein detected between animal samples within a single time point was observed. This may be due in part to varying i.p. injection absorption and metabolic rates among animals. Consistent with immunoblotting, the HA tag of TAT-UCH-L1 was also detected by immunostaining in cortex within Neu-N positive cells in animals sacrificed 4 h post injury. TAT-UCH-L1 was more abundantly detected in peri-injury ipsilateral cortex than the contralateral cortex (Fig 1C). These data indicate that the TAT-UCH-L1 protein transduces neurons in mouse brain *in vivo*.

### TAT-UCH-L1 treatment increases cell survival and preserves brain volume after TBI

**Fig 2:**
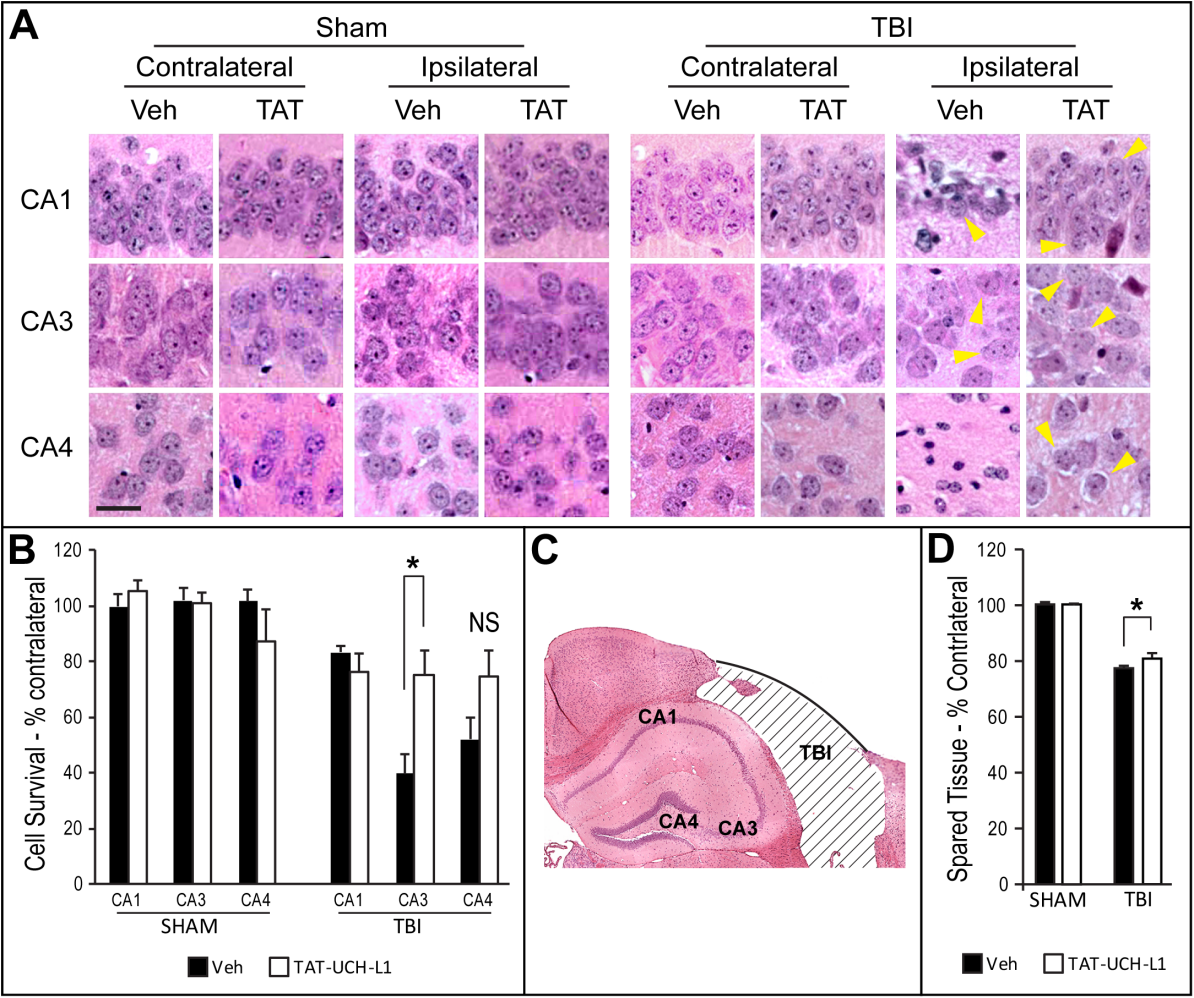
TAT-UCH-L1 treatment increases cell survival and preserves brain volume after TBI. Mice were treated with 60 mg/kg i.p.TAT-UCH-L1 (TAT) or Vehicle (Veh, 1XPBS) 1 h prior to TBI or sham surgery. At post injury day 21, mice were sacrificed via perfusion fixation and brains processed for hematoxylin and eosin (H&E) staining. (A) Representative brightfield photos taken at 20X in regions shown in panel C. Bar = 25 μm. Yellow arrows: illustrative cells with normal morphology. (B) All cells exhibiting normal morphology were counted in CA1, CA3 and CA4 regions of hippocampus at the center of the contusion (Bregma -2.2). Data is shown as percent contralateral. (C) Representative H&E-stained ipsilateral Veh-treated hemisphere. (D) Spared tissue volume expressed as percent contralateral. N = 6 per group. Data are means +/- SE. * P < 0.05. NS: not significant.

To examine whether treatment with TAT-UCH-L1 preserves cell viability and cortical contusion-induced tissue loss after TBI, mice were injected i.p. with 60 mg/kg of TAT-UCH-L1 or vehicle (PBS) 1h before TBI or sham surgery. TAT-UCH-L1- or vehicle- treated mice were sacrificed 21 days post CCI or sham surgery and the brains perfused. Neurons exhibiting normal morphology (yellow arrows) with visible nucleoli were counted in ipsilateral and contralateral CA1, CA3 and CA4 regions of hippocampus (Fig 2A). Cell survival was significantly increased in the CA3 region of TAT-UCH-L1-treated TBI mice compared to vehicle-treated TBI mice (39.59 ± 6.94% vs. 75.01 ± 8.93%) although there was no difference in CA1 (83.21 ± 2.47% vs. 76.36 ± 6.77%) (Fig 2B). Cell survival was also increased in the CA4 region in the TAT-UCH-L1-treated TBI mice as compared to vehicle-treated TBI mice but this effect did not reach statistical significance (51.90 ± 7.84% vs 74.65 ± 9.50%). All sham surgery animals treated with either TAT-UCH-L1 or vehicle exhibited approximately 100% cell survival in CA1, CA3 and CA4 regions.

For spared tissue volume analysis, serial sections were cut and percent contralateral spared tissue volume was measured. As shown in Fig 2D, treatment with TAT-UCH-L1 modestly but significantly preserved spared tissue volume after TBI as compared to the vehicle-treated group (77.74 ± 0.76% vs. 81.22 ± 1.64%). There was no difference between sham TAT-UCH-L1-treated and sham vehicle-treated spared tissue volumes (100.72 ± 0.79% vs. 100.31 ± 0.63%, respectively). Together, the above data demonstrate a protective effect of TAT-UCH-L1 treatment on cell loss after traumatic brain injury.

### TAT-UCH-L1 treatment attenuates APP accumulation and axonal injury after TBI

**Fig 3:**
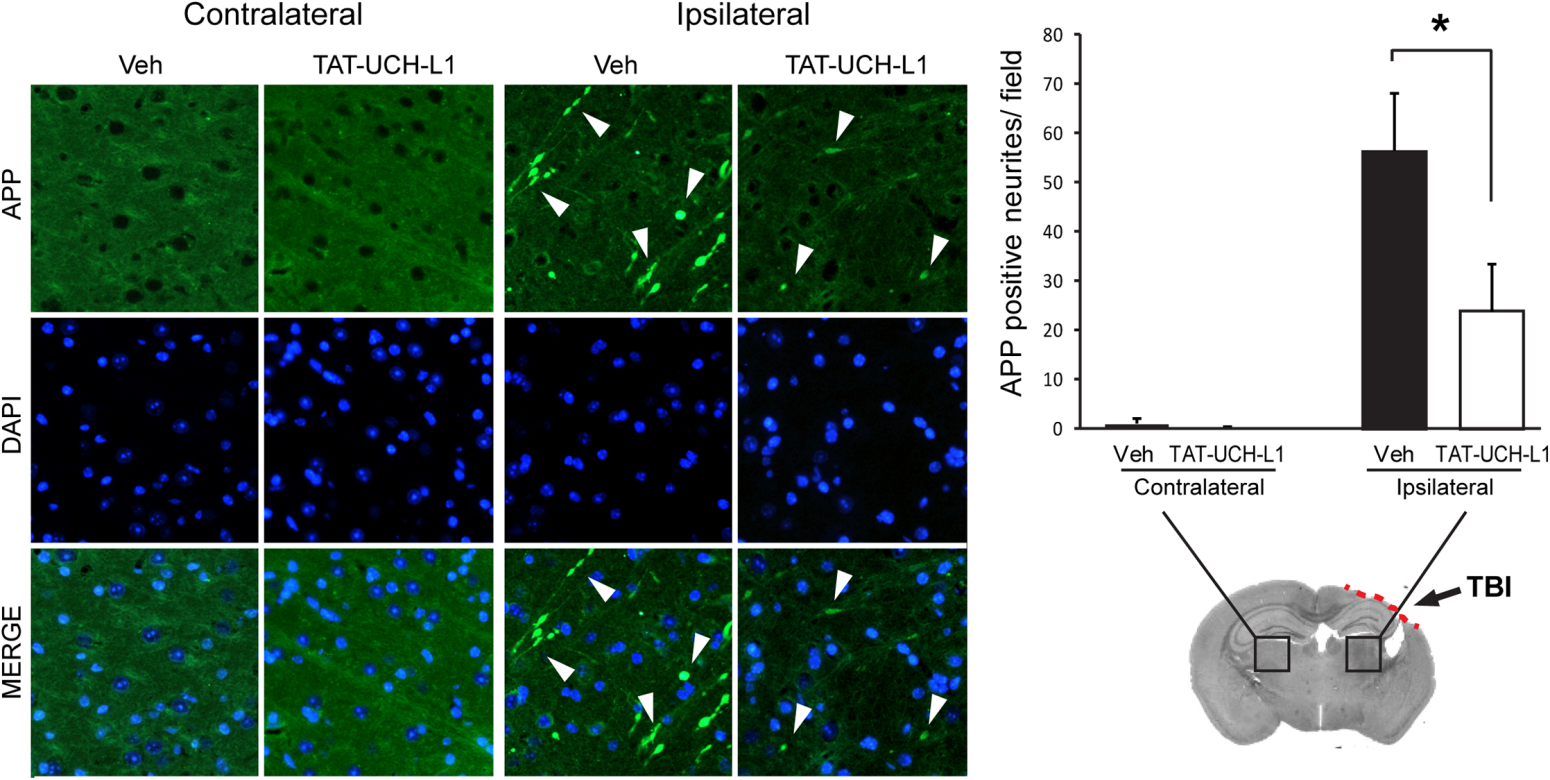
TAT-UCH-L1 treatment attenuates APP accumulation and axonal injury 24h after TBI. Mice were treated with 60 mg/kg TAT-UCH-L1 fusion protein (TAT-UCH-L1) or vehicle (1XPBS, Veh) one hour prior to TBI. Mice were sacrificed via perfusion fixation 24 h after injury and brains processed for anti-APP immunohistochemical analysis. Left: representative fluorophotomicrographs taken at 20X. APP-positive cells (green) are shown at white arrows. Blue is dapi nuclear stain. Right: APP positive neurites per field. N = 5/group. Data are means +/- SE. * P < 0.05 with Bonferroni post hoc analysis.

**Fig 4:**
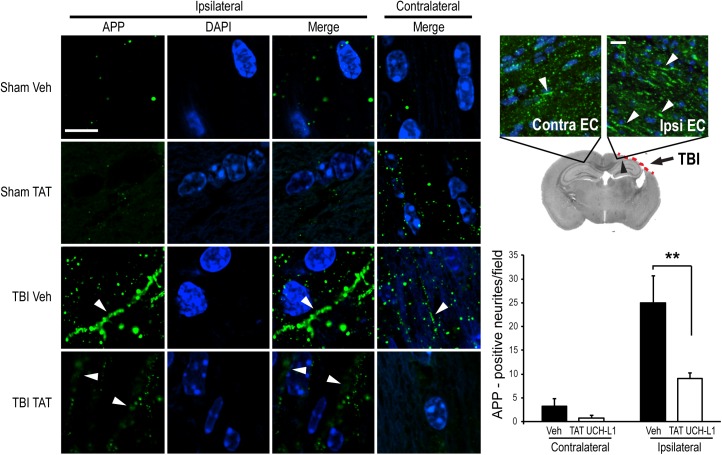
TAT-UCH-L1 treatment attenuates the accumulation of APP in neurites 21 D post traumatic brain injury (TBI). Male mice were injected i.p. with 60 mg/kg TAT- UCH-L1 fusion protein (TAT) or vehicle (Veh) 1 hour prior to TBI or sham surgery. Mice were sacrificed at 21 D post injury and brains perfusion fixed and embedded in paraffin. Left: Representative confocal images of perilesional external capsule (EC) white matter after immunostaining with anti-APP antibody (green). Blue is dapi nuclear stain. White arrows: APP accumulation. Bar = 20 μm. Right *upper*: Representative 20X image of EC in TBI vehicle-treated mouse. Right lower: APP positive neurites per field. N = 5 per group. Data are means +/- SE. ** < 0.01 with Bonferroni post hoc analysis.

To detect the effect of TAT-UCH-L1 on TBI-induced APP accumulation, mice were treated with 60 mg/kg TAT-UCH-L1 or vehicle 1 h prior to TBI and sacrificed by perfusion fixation 24 h after trauma. Brain sections taken at the center of the contusion in trauma- and sham- operated mice were immunostained. APP-positive neurites were counted in ipsilateral and contralateral thalamus (Fig 3). APP accumulation was diminished in animals treated with TAT-UCH-L1 as compared to vehicle after injury (11.81 ± 9.55 vs. 56.40 ± 23.80 APP-positive neurites respectively). There were no differences between contralateral regions of post-injury TAT-UCH-L1- and vehicle- treated animals (0.20 ± 0.20 vs. 1.00 ± 1.00 APP-positive neurites respectively). In a separate cohort of animals, mice were treated as described above and sacrificed 21 D post injury. Positive neurites were counted in ipsilateral and contralateral external capsule (Fig 4). Consistent with the above data, APP accumulation was significantly decreased in animals treated with TAT-UCH-L1 as compared to vehicle (25.00 ± 5.64 vs. 9.00 ± 1.18 APP-positive neurites respectively). Therefore, the above data suggests that treatment with TAT-UCH-L1 attenuates TBI-induced axonal injury.

### TAT-UCH-L1 treatment decreases Ub-protein accumulation and autophagy activation after TBI

**Fig 5:**
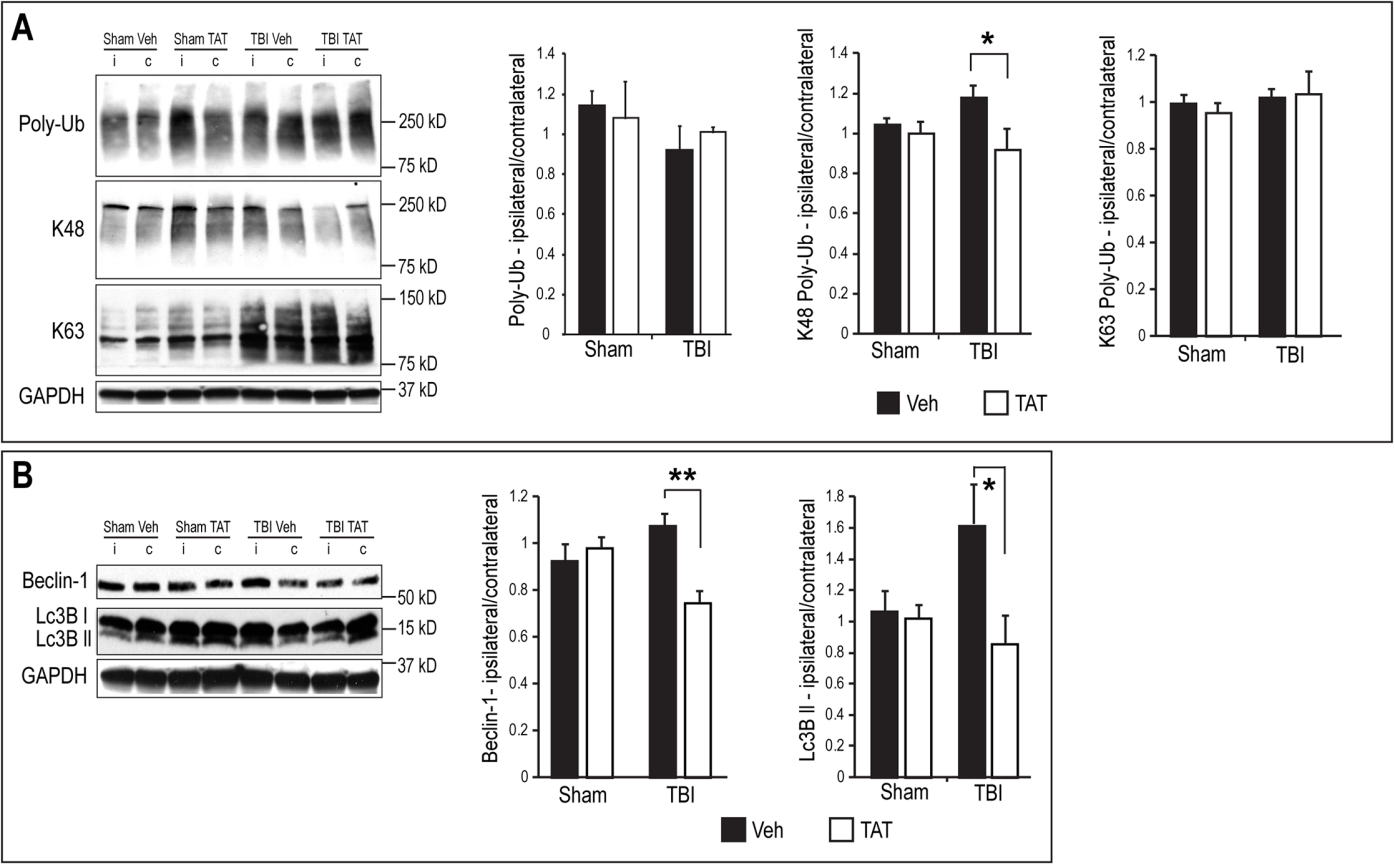
TAT-UCH-L1 treatment attenuates TBI-induced increase in K48-linkage poly-Ub protein accumulation and autophagy activation in hippocampus. Mice were injected i.p. with 60 mg/kg TAT-UCH-L1 (TAT) or vehicle (1XPBS, Veh) then underwent CCI or sham surgery. Mice were sacrificed 24 h post injury and hippocampi removed for immunoblotting with: (A) Anti-poly-Ubiquitin, anti-K48-linkage ubiquitin and anti-K63-linkage ubiquitin antibodies or (B) anti-Beclin-1 and anti-LC3B antibodies. GAPDH was used as a loading control. Left: representative immunoblots; right: Densitometric analysis of immunoblots expressed as ipsilateral/contralateral. N = 5 per group. Data are means +/- SE. * P < 0.05, ** P < 0.01 with Dunnett’s post hoc analysis.

To detect the effect of TAT-UCH-L1 treatment on TBI-induced Ub-protein accumulation and autophagy activation, a separate cohort of animals underwent trauma or sham surgery and was sacrificed at 24 h. Poly-Ub proteins and the autophagy markers, Beclin-1 and LC3B, were detected in hippocampus with immunoblotting. As shown in Fig 5A, no significant change in poly-Ub protein levels was observed between trauma animals treated with TAT-UCH-L1 and vehicle-treated controls (1.01 ± 0.03 vs. 0.91 ± 0.18, P>0.05), or trauma animals compared to sham animals in either group. Next, specific modification of proteins by K48-linked poly-Ub and K63-linked poly-Ub was detected using anti-K48-poly-Ub and anti-K63-poly-Ub antibodies. As shown in Fig 5A, TBI was associated with increased accumulation of K48-linked poly-Ub proteins in vehicle-treated mice and this increase was abolished in TAT-UCH-L1-treated mice (1.16 ± 0.06 vs. 0.91 ± 0.11, P<0.05). In contrast to hippocampal K48-linked poly-Ub levels, TBI did not result in a change in hippocampal expression of K63-linked poly-Ub. TAT-UCH-L1 treatment had no significant effect on K63-linked poly-Ub in either sham or TBI groups.

Hippocampal protein levels of both beclin-1 and LC3BII were increased in vehicle-treated mice 24 h after TBI as compared to sham mice. Compared with vehicle-treated TBI mice, TAT-UCH-L1 treatment significantly decreased both beclin-1 (1.07 ± 0.04 vs. 0.75 ± 0.05, P<0.01) and LC3BII levels (1.61 ± 0.37 vs. 0.86 ± 0.18, P<0.05), suggesting that TAT-UCH-L1 treatment attenuates autophagy activation induced by TBI (Fig 5B).

## Discussion

The major findings of the study are: 1) Modification of UCH-L1 by fusion of the protein with the PTD of the TAT protein produces a systemically active protein that transduces brain neurons *in vivo*. 2) Treatment with TAT-UCH-L1 decreases concentrations of K48-linked poly-Ub proteins after TBI and attenuates increases in Beclin-1 and LC3BII. 3) TAT-UCH-L1 treatment decreases accumulation of APP in damaged axons and increases cell survival in CA3 after TBI.

The PTD domain of TAT confers neuronal permeability to the HIV virus. This sequence has been exploited by construction of several TAT proteins that cross the blood brain barrier and readily transduce neurons *in vivo*. Several such constructs have been used in animal models of neurological diseases including: Fusion of the PTD of TAT with the anti-apoptotic protein Bcl-XL produces a brain permeable protein that decreases activation of caspase 3, reduces infarction volume and improves motor outcomes after temporary focal ischemia in mice[27]; A TAT 14-3-3ε fusion protein reduced autophagy and improved histological outcome and motor function after ischemia in mice [28]; A TAT fusion protein, TAT-NR2B9c, also named NA-1, which comprises nine C-terminal residues of NR2B, an inhibitor of a postsynaptic scaffolding protein (PSD-95 protein), transduces neurons when given systemically, has neuroprotective effects and is well tolerated in human studies [29]. Thus, TAT-proteins have excellent potential for human translation.

UCH-L1 is expressed almost exclusively in neurons. The protein is highly prevalent, constituting as much as 1% of brain protein, hence the protein may have a role in many neuron-specific functions[8]. UCH-L1 may possess a number of activities and physiological functions. It can both ligate Ub to proteins to form poly-Ub chains and it can also hydrolyze Ub from proteins [7]. UCH-L1 also binds to and stabilizes monoubiquitin in neurons [30]. Tagging proteins with Ub is most commonly associated with transport of misfolded proteins to the proteasome for degradation in the UPP [10]. When proteins reach the proteasome, Ub is hydrolyzed from the protein so the misfolded protein can enter the proteasome and the Ub can be recycled. UCH-L1, which can both ligate and hydrolyze Ub, has been proposed to be a significant component of the neuronal UPP. UCH-L1 dysfunction has been associated with the accumulation of Ub-proteins and inhibition of autophagy in different cells [19, 22, 31, 32]. In the current study we found that treatment with TAT-UCH-L1 did not produce a significant change in protein ubiquitination after TBI as detected by a poly-Ub antibody or the K63-linked poly-Ub antibody. However, treatment with TAT-UCH-L1 significantly attenuated post-injury increase of K48-linked poly-Ub. K48-linked poly-Ub acts as a universal indicator for proteins targeted for proteasomal degradation while the K63 poly-Ub linkage is not associated with proteasome degradation and may play a role in endosome transport and other intracellular functions [5]. These results suggest that TAT-UCH-L1 treatment enhances protein degradation through the UPP since the K48-Ub protein modification is associated with protein clearance[33]. We also found that CA3 neuronal cell survival was significantly increased and spared brain tissue volume was significantly higher in TAT-UCH-L1-treated mice. Since accumulation of unfolded proteins may interfere with normal cell function and induce neuronal stress, TAT-UCH-L1’s enhancement of neuronal UPP function may also increase neuronal cell survival after TBI.

Clearance of misfolded and damaged proteins from neurons is also mediated by autophagy in addition to the UPP[34]. UCH-L1 may also regulate autophagy, and a mutation in UCH-L1 associated with familial Parkinson’s disease may inhibit chaperone-mediated autophagy by the interaction with LAMP2A, Hsc70, Hsp90 and increase the accumulation of α-synuclein [31]. UCH-L1 dysfunction also has been reported to excerabate the human-IAPP- induced defect in the autophagy/lysosomal pathway in β-cells [32]. Our results demonstrate that TAT-UCH-L1 treatment decreases beclin-1 and LC3BII after TBI, consistent with attenuated activation of autophagy. This contradictory result may be explained by increased clearance of damaged and misfolded proteins through the UPP after treatment with TAT-UCH-L1 rather than a direct effect of TAT-UCH-L1 on autophagy.

In addition to UCH-L1’s role in the neuronal UPP, it may have other functions. Many of the components of the UPP found in other cell types are present in the neuron; thus UCH-L1 may not only be required for UPP function. UCH-L1 may also be important in other neuron-specific transport functions. The neuron has unique requirements for transport of proteins and organelles from the soma down the axon to maintain the integrity of the axon. UCH-L1 interacts with cytoskeletal proteins such as tubulin and neurofilaments [35, 36]. UCH-L1 mutations and disruption of the gene produce major axonal and dendritic pathology. The GAD mouse bearing a nonsense mutation in UCH-L1 is characterized by 'dying-back'-type axonal degeneration, axonal swelling and formation of spheroid bodies in nerve terminals within the brain [37, 38]. A human mutation in UCH-L1 has been identified that produces extensive white matter changes and neurodegeneration [16]. UCH-L1 has been reported to regulate brain-derived neurotrophic factor-dependent retrograde transport [12]. Thus UCH-L1 may be required for normal axonal structure and function.

There is also evidence that UCH-L1 is important in synaptic function. UCH-L1 expression is regulated by synaptic activity and alters synaptic protein distribution, thus UCH-L1 may play an important role in synaptic function and remodeling [39]. Inhibition of UCH-L1 by LDN57444 blocks long term potentiation in hippocampal slices [9]. The UCH-L1 null mouse has been shown to have impaired synaptic transmission and loss of normal synaptic vesicles accompanied by accumulation of abnormal structures in the presynaptic nerve terminal [39, 40]. Furthermore, UCH-L1 null mice have disintegration of the apical dendrite and loss of dendritic spines resulting in motor neuron dysfunction in brain [41]. These and other results suggest that UCH-L1 is essential for synaptic function and may play a vital role in remodeling synapses, an important mechanism for recovery after TBI.

The current results support a role for UCH-L1 in maintaining and repairing damaged axons after TBI. APP is actively transported down axons and also has a role in axonal transport itself [10]. APP accumulates at the site of axonal injury and thus is commonly used as a marker of axonal injury after TBI [3, 4, 10, 42]. We found that TAT-UCH-L1-treated mice had less axonal damage as detected by accumulation of APP in damaged axons in thalamus 24h after controlled cortical impact. In addition there was less evidence of axonal damage in the external capsule 21 days after TBI. These results suggest that the restoration of UCH-L1 activity by TAT-UCH-L1 treatment preserves axonal integrity, perhaps by improving transport of vital proteins down the axons. In addition, TAT-UCH-L1 preserved cell viability in hippocampus, possibly by preventing apoptosis due to Wallerian degeneration of axons.

The current study is the first to show that TAT-UCH-L1 treatment may reduce histological damage and axonal integrity after TBI. There are several limitations to this study: These studies use only pretreatment paradigms and do not assess whether the treatment has an effect on cognitive or motor function. The studies provide limited data regarding the mechanisms by which TAT-UCH-L1 prevents axonal damage and cell death after TBI. These studies do not address long term effects on axonal pathology, neuronal cell death and function outcome; therefore, additional studies are needed. Despite these limitations, the current work demonstrates the promise of TAT-UCH-L1 for the treatment of axonal injury after TBI. In order to optimize TAT-UCH-L1 efficacy, pharmacokinetic studies are needed to design an optimal dosing paradigm. In addition, further modification of the protein construct such as mutation of C152A or truncation of the N-terminus may enhance potency and half-life of the TAT protein [22, 43, 44]. Additional studies are required to further investigate the mechanisms by which TAT-UCH-L1 affords protection including identifying the direct molecular targets of UCH-L1. The translational potential of the approach will require administering the TAT-UCH-L1 at various time windows after TBI, and assessing whether the treatment improves motor and cognitive behavior. Gong et al have shown that TAT-UCH-L1 treatment can restore LTP *in vitro* and improve memory function in a mouse model of Alzheimer’s disease [9]. Thus, TAT-UCH-L1 has the potential to improve cognitive function when administered weeks or months after TBI. Furthermore, it will be informative to determine the effect of TAT-UCH-L1 treatment upon injury biomarkers such as GFAP and serum UCH-L1[45]. Thus, many additional studies are needed to investigate the administration of TAT-UCH-L1 in the treatment of TBI.

## Supporting information

S1 Fig Sequence results for TAT-UCH-L1 WT/pET 30. (DOCX)

S2 Fig UCH-L1 hydrolase activity is not altered in TAT fusion protein. 100nM of recombinant TAT-UCH-L1 WT, TAT -UCH-L1 C90S fusion proteins or recombinant UCH-L1 WT or C90S proteins were incubated with 500 nM Ubiquitin-AMC substrate and hydrolase activity was measured by detecting fluorescence intensity (arbitrary fluorescence units) generated by the cleavage of Ubiquitin-AMC. n = 4 per group. Data is expressed as means +/- SE.
(DOC)

S1 Text Supplementary data. (DOCX)
