# Supplementary material for: In vivo transduction of neurons with TAT-UCH-L1 protects brain against controlled cortical impact injury
Source: PLoS One. 2017 May 24;12(5):e0178049. doi: 10.1371/journal.pone.0178049 (PMC5443532; doi:10.1371/journal.pone.0178049)
Supplement: S1 Fig — (DOCX) [file pone.0178049.s001.docx]

**TAT-UCHL1 WT/pET 30 Sequence Results**

ATANCNANTCCCCNTCTNNAAANNANTNNGTTANNNNNAAGAAGGANANNNNCATATGAA

A**CACCATCATCATCATCATTCTGGTCTGGTGCCACGCGGTTCTTATGGCCGCAAAAAACG**

**CCGACAGCGCCGACGCTATGGTTATCCGTACGACGTTCCGGACTACGCT**GATATCATGCA

GCTGAAACCGATGGAGATTAACCCCGAGATGCTGAACAAAGTGTTGGCCAAGCTGGGGGT

CGCCGGGCAGTGGCGCTTTGCCGACGTGCTAGGGCTGGAGGAGGAGACTCTGGGCTCAGT

GCCATCTCCTGCCTGCGCCCTGCTGCTGCTGTTTCCCCTCACGGCCCAGCATGAAAACTT

CAGGAAAAAACAAATTGAGGAACTGAAGGGACAAGAAGTTAGCCCTAAAGTTTACTTCAT

GAAGCAGACCATCGGGAACTCCTGTGGTACCATTGGGCTGATCCACGCAGTGGCCAATAA

CCAAGACAAGCTGGAATTTGAGGATGGATCAGTCCTGAAACAGTTTCTGTCTGAAACGGA

GAAGTTGTCCCCTGAAGACAGAGCCAAGTGTTTCGAGAAGAACGAGGCCATTCAGGCAGC

CCATGACTCCGTGGCCCAGGAGGGCCAGTGCCGGGTAGACGACAAAGTGAATTTCCATTT

TATCCTGTTCAATAATGTGGACGGCCACCTCTACGAGCTCGATGGGCGAATGCCTTTCCC

CGTGAACCATGGCGCCAGTTCAGAGGACTCTCTGCTGCAGGATGCCGCCAAGGTCTGCAG

AGAATTCACTGAGCGCGAGCAGGGAGAGGTCCGCTTCTCCGCAGTGGCTCTCTGCAAAGC

AGCCTAACTCGAGCACCACCACCACCACCACTGAGATCCGGCTGCTAACAAA

**Purple Font:** 6 X His sequence

**Orange Font:** Thrombin sequence

**Blue Font:**  PTD sequence

**Green Font:**  HA sequence

Red Font: first and last codon for the UCHL1 sequence

Yellow highlighting: Restriction sites
