# Supplementary figures and images for: In vivo transduction of neurons with TAT-UCH-L1 protects brain against controlled cortical impact injury
Source: PLoS One. 2017 May 24;12(5):e0178049. doi: 10.1371/journal.pone.0178049 (PMC5443532; doi:10.1371/journal.pone.0178049)

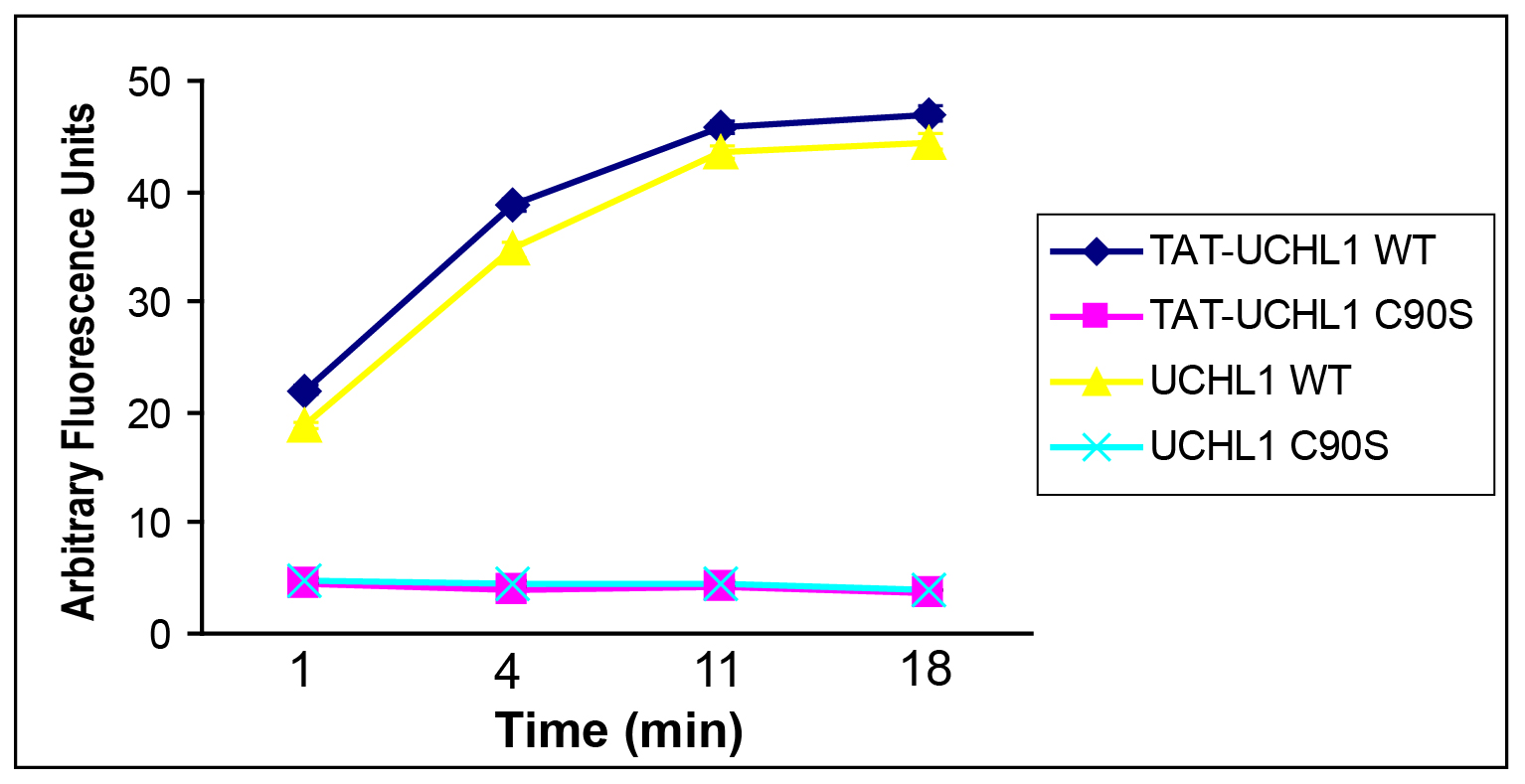

Supplement: S2 Fig — 100nM of recombinant TAT-UCH-L1 WT, TAT -UCH-L1 C90S fusion proteins or recombinant UCH-L1 WT or C90S proteins were incubated with 500 nM Ubiquitin-AMC substrate and hydrolase activity was measured by detecting fluorescence intensity (arbitrary fluorescence units) generated by the cleavage of Ubiquitin-AMC. n = 4 per group. Data is expressed as means +/- SE. (DOC) [file pone.0178049.s002.doc]
