# Supplementary material for: In vivo transduction of neurons with TAT-UCH-L1 protects brain against controlled cortical impact injury
Source: PLoS One. 2017 May 24;12(5):e0178049. doi: 10.1371/journal.pone.0178049 (PMC5443532; doi:10.1371/journal.pone.0178049)
Supplement: S1 Text — (DOCX) [file pone.0178049.s003.docx]

**Supplementary Data:**

**Materials and methods:**

**Hydrolase activity assay**

The UCHL1 hydrolase activity assay was performed as previously described [[1](#_ENREF_1),[2](#_ENREF_2)]. Briefly, 100nM of recombinant TAT-UCHL1 wild-type (WT), TAT -UCH-L1 C90S fusion proteins or recombinant UCHL1 WT or C90S proteins were incubated with 500 nM Ubiquitin-AMC substrate (BostonBiochem, Cambridge,MA) in hydrolase buffer. Free AMC fluorophore generated by the cleavage of Ubiquitin-AMC was read using a fluorescent plate reader (ex 360nm, em 460 nm). n = 4 per group. Data is expressed as means +/- SE.

1. Liu H, Li W, Ahmad M, Miller TM, Rose ME, et al. (2011) Modification of ubiquitin-C-terminal hydrolase-L1 by cyclopentenone prostaglandins exacerbates hypoxic injury. Neurobiol Dis 41: 318-328.

2. Liu H, Li W, Rose ME, Hickey RW, Chen J, et al. (2015) The point mutation UCH-L1 C152A protects primary neurons against cyclopentenone prostaglandin-induced cytotoxicity: implications for post-ischemic neuronal injury. Cell Death Dis 6: e1966.
